# The OptimaMed intervention to reduce inappropriate medications in nursing home residents with severe dementia: results from a quasi-experimental feasibility pilot study

**DOI:** 10.1186/s12877-018-0895-z

**Published:** 2018-09-04

**Authors:** Machelle Wilchesky, Gerhard Mueller, Michèle Morin, Martine Marcotte, Philippe Voyer, Michèle Aubin, Pierre-Hugues Carmichael, Nathalie Champoux, Johanne Monette, Anik Giguère, Pierre Durand, René Verreault, Marcel Arcand, Edeltraut Kröger

**Affiliations:** 1 Department of Family Medicine and Division of Geriatric Medicine, McGill University, 5858, Chemin de la Côte-des-Neiges, Montreal, Quebec H3S 1Z1 Canada; 2 Donald Berman Maimonides Centre for Research in Aging, 5795 Caldwell Avenue, Montreal, Quebec H4W 1W3 Canada; 3 Department of Nursing Science and Gerontology, UMIT-The Health & Life Sciences University, Eduard-Wallnoefer-Zentrum 1, A-6060 Hall in Tyrol, Tyrol Austria; 4 Centre d’excellence sur le vieillissement de Québec, Centre intégré universitaire de santé et de services sociaux de la Capitale-Nationale, 1050, Chemin Ste-Foy, room L2-30, Quebec City, Quebec G1S 4L8 Canada; 5 Laval University, 1050, avenue de la Médecine, Quebec City, Quebec G1V 0A6 Canada; 6 Faculté de médecine, Université de Montréal, 2900 Boulevard Edouard-Montpetit, Montreal, Quebec H3T 1J4 Canada; 7 Division of Geriatric Medicine, McGill University, Jewish General Hospital, 3755 Côte-Ste-Catherine, Montreal, Quebec H3T 1E2 Canada; 8 Centre de recherche sur le vieillissement, affilié à l’Université de Sherbrooke, 1036, rue Belvédère Sud, Sherbrooke, Quebec J1H 4C4 Canada

**Keywords:** Intervention, Inappropriate medication use, Long-term care, Dementia

## Abstract

**Background:**

Medication regimens in nursing home (NH) residents with severe dementia should be frequently reviewed to avoid inappropriate medication, overtreatment and adverse drug events, within a comfort care approach. This study aimed at testing the feasibility of an interdisciplinary knowledge exchange (KE) intervention using a medication review guidance tool categorizing medications as either “generally”, “sometimes” or “exceptionally” appropriate for NH residents with severe dementia.

**Methods:**

A quasi-experimental feasibility pilot study with 44 participating residents aged 65 years or over with severe dementia was carried out in three NH in Quebec City, Canada. The intervention comprised an information leaflet for residents’ families, a 90-min KE session for NH general practitioners (GP), pharmacists and nurses focusing on the medication review guidance tool, a medication review by the pharmacists for participating residents with ensuing team discussion on medication changes, and a post-intervention KE session to obtain feedback from team staff. Medication regimens and levels of pain and of agitation of the participants were evaluated at baseline and at 4 months post-intervention. A questionnaire for team staff explored perceived barriers and facilitators. Statistical differences in measures comparing pre and post-intervention were assessed using paired t-tests and Cochran’s-Q tests.

**Results:**

The KE sessions reached 34 NH team staff (5 GP, 4 pharmacists, 6 heads of care unit and 19 staff nurses). Forty-four residents participated in the study and were followed for a mean of 104 days. The total number of regular medications was 372 pre and 327 post-intervention. The mean number of regular medications per resident was 7.86 pre and 6.81 post-intervention. The odds ratios estimating the risks of using any regular medication or a “sometimes appropriate” medication post-intervention were 0.81 (95% CI: 0.71–0.92) and 0.83 (95% CI: 0.74–0.94), respectively.

**Conclusion:**

A simple KE intervention using a medication review guidance tool categorizing medications as being either “generally”, “sometimes” or “exceptionally” appropriate in severe dementia was well received and accompanied by an overall reduction in medication use by NH residents with severe dementia. Levels of agitation were unaffected and there was no clinically significant changes in levels of pain. Staff feedback provided opportunities to improve the intervention.

**Electronic supplementary material:**

The online version of this article (10.1186/s12877-018-0895-z) contains supplementary material, which is available to authorized users.

## Background

Medication use is considered optimal when the prescribed medications are well tolerated and have a clear indication based on scientific evidence. Age-related physiological changes, however, may result in altered pharmacokinetic and pharmacodynamic responses to medications, thereby reducing their tolerability in older patients [1]. Moreover, some commonly prescribed medications offer limited benefit in the face of shortened life expectancy [2–4]. Medications presenting unfavorable adverse event risk to benefit ratios are associated with negative health outcomes [5–8]. Prevalence of potentially inappropriate medication prescriptions to seniors (aged 65 and over) is estimated as being high [9–11]. Furthermore, seniors with dementia, who may be incapable of verbalizing symptoms associated with adverse drug effects [12] are at even greater risk of inappropriate prescribing [3, 13–16].

Optimal medication use in seniors with severe dementia residing in nursing homes (NHs) presents an ongoing challenge. Health professionals, who may not always acknowledge severe dementia as being a terminal disease, may expose patients to unnecessarily aggressive treatments [17, 18]. Medication regimens in these patients should be frequently reconsidered to take into account changes in patients’ condition, avoid overtreatment and adverse drug events, and improve symptom control and comfort [15]. A body of research has produced guidance for medication appropriateness in seniors, most notably the Beers criteria [19] and the STOPP/START consensus [20]. While these lists indicate medications that are inappropriate for seniors, they do not, however, specifically address the issue of medication appropriateness for seniors with severe dementia who are even more vulnerable and have shortened life expectancy, like those living in NHs.

Building upon prior research that did address this issue in patients with severe dementia [21, 22], our previous study engaged a panel of experts to categorize medications that are of questionable benefit for Quebec NH seniors with severe dementia [23]. Briefly, the 15-member multidisciplinary Delphi panel agreed on the categorization of 63 medications or medication classes as being either “generally”, “sometimes,” or “exceptionally” appropriate for these patients as shown in Additional file 1*.* The aim of the present pilot study was to test the feasibility of an interdisciplinary knowledge exchange (KE) intervention using this list, and to measure its impact on medication use, and on pain and agitation levels in this population.

## Methods

### Study design

A quasi-experimental (pre-post) study was conducted within three Quebec City NHs between January and December 2014.

### Setting

In Canada, public NHs are financed at the provincial level, leading to differences in their organization and management of care across the country. In the province of Quebec, general practitioners (GPs) and clinical pharmacists work on a part-time basis in public NHs that provide 24-h nursing care for people with complex needs. The present study was proposed to the local Health and Social Services Board (HSSB), which suggested the name of three NHs that agreed to participate. The study was approved by the HSSB and the *CHU de Québec* research centre ethics review boards (Ethics Certificate # C13-12-1886 / 2013–2014-25).

### Participant eligibility and recruitment procedures

To be eligible, residents within the three participating NHs had to be 65 years of age or older, have a diagnosis of severe dementia (of any etiology) recorded within their medical chart, and have resided in this NH for at least 2 months. The level of dementia severity is not usually available in the NH medical chart therefore, the Functional Autonomy Measurement System (*Système de Mesure de l’Autonomie Fonctionnelle*, SMAF) was used as a proxy measure [24]. SMAF is a broadly validated tool that predicts the needs of seniors and disabled persons on the basis of the WHO’s classification of impairments, disabilities and handicaps. It measures performance on 29 functions of 1) activities of daily living, 2) mobility, 3) communication, 4) mental functions, and 5) instrumental activities of daily living. This leads to a numeric Iso-SMAF profile, which is used since 2005 to assess admission eligibility to Quebec NHs. Residents with Iso-SMAF profiles 13 and 14 (corresponding to stage 7 on the Reisberg FAST scale [25]) were included for study. There was no Iso-SMAF profile on record for three potentially eligible residents admitted before 2005, these residents Iso-SMAF profile was therefore evaluated by the NH nurses directly involved with their care.

**Fig. 1:**
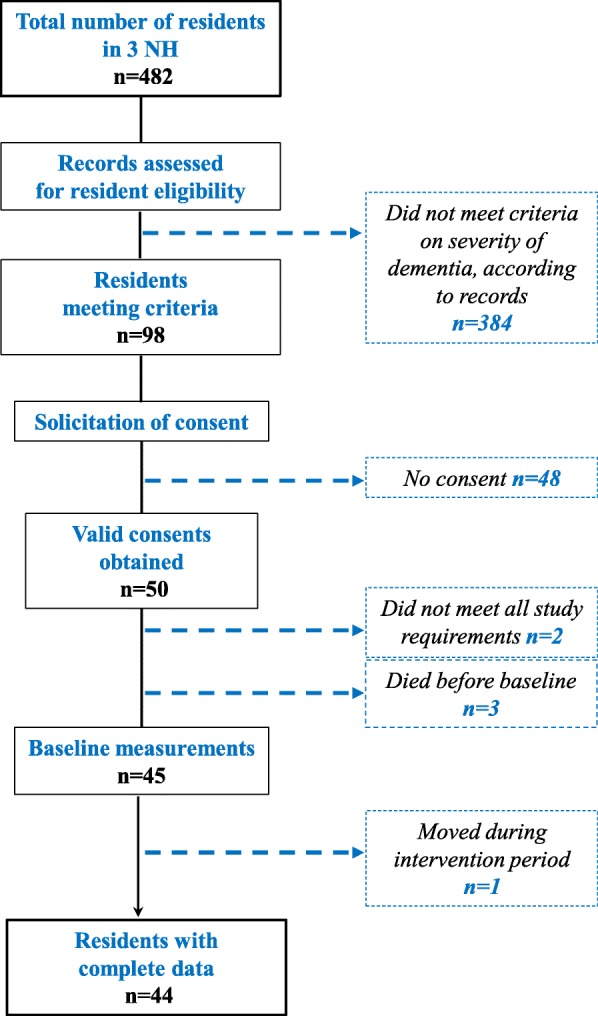
Flowchart for the selection of participating residents

Letters of invitation to participate in the study, along with a two-page informational leaflet about medication use in seniors with severe dementia, were sent to the families or legal guardians of eligible residents. A flow chart depicting study recruitment procedures is presented in Fig. 1.

### Medication review guidance

A medication review guidance (MRG) tool was developed from the medication appropriateness list agreed upon by the Delphi panel [23], for use in the province of Quebec NHs. In addition to being in French, the tool uses the American Hospital Formulary Service (AHFS) medication classes, and examples of drugs used in Quebec NHs as well as summary explanations (available at: http://www.ciusss-capitalenationale.gouv.qc.ca/sites/default/files/medication_demence_severe_oct2015.pdf).

### Knowledge exchange intervention

An interdisciplinary 90-min continuous education and Knowledge Exchange (KE) session, held by a geriatrician with extensive experience in continuous education (MMorin) and a pharmacist (EK), was conducted at each participating institution and included the institution’s physicians, nurses and pharmacists. First, the context of the study as well as issues pertaining to the complexity of prescribing for seniors with severe dementia (e.g. frailty, multimorbidity, polypharmacy, metabolic changes) were presented. Second, the MRG tool (summarized in Additional file 1) and its intended use were explained. Finally, a clinical vignette representing a typical NH resident was used to illustrate how the MRG tool could facilitate medication reviews and the ensuing discussions.

Participating NH pharmacists were then asked to perform a medication review, using the MRG tool provided, for all study residents. The ensuing recommendations would be discussed with families and during NH multidisciplinary meetings involving the treating physician and, whenever possible, the treating nurse. The final decision regarding changes in medication regimen would be taken by each resident’s physician, applying clinical judgment and considering all relevant information on the resident’s clinical, psychological and social circumstances, level of care, and family considerations. The clinical decision and deprescribing processes were left to the care team discretion.

Finally, a second KE session was held at the end of the follow-up period where all participating health professionals were asked to complete a questionnaire and to provide feedback for the purposes of intervention improvement.

### Measurements

Data pertaining to age, sex and medical comorbidities [26] were collected from medical records of participating residents at baseline. The Observation Grid for Medication-Taking [27] was used by the study nurse to assess problems with medication taking in participating residents.

Baseline and post follow-up medication use were derived from the NH pharmacy database, and each current (“active”) prescription per participating resident was categorized according to the appropriateness list presented in Additional file 1 [23].

In order to evaluate whether deprescribing as a result of our intervention had resulted in adverse effects, levels of pain and of agitation were measured in participating residents during both the pre and post-intervention periods. Levels of pain and agitation were assessed during a total of four fifteen-minute observation periods, both at rest and during mobilization, and on two different days. The 60 items from the French version of the Pain Assessment Checklist for Seniors with Limited Ability to Communicate (PACSLAC-F) were used to evaluate the level of pain [28, 29]. PACSLAC-F, already used in many NHs, is based on the observation of residents’ facial expression, activity or body movement, social behaviour, personality or mood, physiological changes or changes in eating, sleeping or vocal behaviour. No cut-off has been defined for this tool. Levels of agitation were measured by the total number of times any Cohen-Mansfield Agitation Inventory (CMAI) item was observed during the observation periods and scores over 45 should reflect severe agitation [30, 31]. Whenever possible, multidisciplinary meetings were attended by the study nurse to document discussions surrounding the pharmacist recommendations.

Finally, intervention relevance and feasibility were evaluated by NH GPs, pharmacists and nurses attending the post-intervention KE session via completion of a semi-structured questionnaire that included the opportunity to respond to open-ended questions.

### Data analyses

Descriptive statistics were computed for all variables collected at baseline (Table 1) and for all resident outcomes for both the pre and post-intervention periods (Table 2). Statistical differences in measures comparing pre and post-intervention were assessed using paired t-tests and Cochran’s-Q tests for continuous and categorical outcomes, respectively. Odds ratios modeled using generalized linear mixed models (GLMM) were used to estimate the odds of having a prescription within each of the 3 medication appropriateness categories comparing the pre vs. post-intervention period. The GLMM model allowed for intra-prescription correlation, but assumed that prescriptions for a given individual were independent. Given the small sample size, no further adjustment could be performed. For similar reasons, the model included only effects for the appropriateness category, time, a category by time interaction, and no adjustment for confounding variables. All analyses were conducted using SAS 9.4.

**Table 1:** Baseline characteristics, *n* = 44 participating residents

	Mean or %	SD
Age (years, mean)	86.9	6.9
Female (%)	70.5	n/a
Follow-up (days, mean)	104	13.5
Medication administration problems (scale of 0 to 13, mean)^a^	0.23	0.86
Charlson Comorbidity Score (mean)	7.45	2.46
Residents with severe agitation (> 45^b^, %)	13.6	12.8
Discomfort/pain (scale of 0 to 60, mean)^c^	8.1	2.3

SD, standard deviation

^a^Total number of problematic behaviors observed during medication administration [27]

^b^Sum of the number of times the Cohen-Mansfield Agitation Inventory items [31] were observed during the four 15-min observation periods

^c^Sum of observations of PACSLAC-F items [29] during the four 15-min observation periods

**Table 2:** Intervention outcomes among 44 participating residents

Medication use	Pre-intervention	Post follow-up	*p*-value^***^
Total number of regular medications (n)	372	327	0.0003
Total number of “generally appropriate” medications (n)	99	112	0.0741
Total number of “sometimes appropriate” medications (n)	194	167	0.0003
Total number of “exceptionally appropriate” medications (n)	12	10	0.4795
Number of medications per participant (mean ± SD)	7.86 ± 3.78	6.82 ± 3.75	0.0007
Proportion of participants using “generally appropriate” medications (%)	90.9	93.2	0.3173
Proportion of participants using “sometimes appropriate” medications (%)	97.7	97.7	1.000
Proportion of participants using “exceptionally appropriate” medications (%)	20.5	18.2	0.5637
Level of agitation (mean ± SD)^*^	21.1 ± 19.5	21.3 ± 15.9	0.7139
Level of pain (scale 0 to 60, mean ± SD)^**^	8.1 ± 2.3	9.7 ± 2.5	< 0.0001

SD, standard deviation

^*^Sum of the number of times the Cohen-Mansfield Agitation Inventory items [31] were observed during the four 15-min observation periods

^**^Sum of observations of PACSLAC-F items [29] during the four 15-min observation periods

^***^p-values were estimated using Cochran’s Q test for categorical variables and paired T-test for continuous variables

## Results

Thirty-four NH health professionals participated in the first KE session: 5 GPs, 4 clinical pharmacists, 6 heads of NH care units (registered nurses or administrators), and 19 staff nurses (registered or auxiliary nurses). A total of 98 residents met the initial eligibility criteria as determined by medical records, and consent for study participation was obtained, from those who were entitled to take care decisions for these residents, for 50 eligible residents. Five potential participants were lost during the pre-intervention period: for two residents meeting the required Iso-SMAF profile, the level of dementia severity turned out to be below stage 7 on the FAST scale; three residents died before the intervention start date. In addition, one patient moved during the intervention period. For all remaining 44 residents, at least one medication review took place during the study period, and medication data both at baseline and at follow-up were available. Five participants died during the follow-up period before their level of pain and of agitation could be assessed. Baseline characteristics of the 44 participating residents are presented in Table 1. The participants have resided in the NH between 2 months and 16 years (mean 4.13 years; SD 3.13).

Mean follow-up for all 44 residents was 104 days (SD 13.5). The study nurse reported that pharmacists revised the medication regimens of all 44 participating residents using the provided medication review guidance tool. Pharmacist recommendations were discussed during scheduled meetings with the responsible GP; however, nurses did not generally attend these meetings but they were consulted as needed. The observation grid for medication-taking [27] did not provide much additional information since administration of crushed tablets mixed with yogurt or fruit purees was already used for residents with dysphagia.

Medication regimens of participating residents included 240 different medications, of which 22 (9%) were considered “generally”, 109 (45%) “sometimes”, and 29 (12%) “exceptionally” appropriate according to the medication appropriateness list (Additional file 1). Seventeen (7%) of the prescribed medications corresponded to those for which the Delphi panel had not been able to reach a consensus, including cholinesterase inhibitors, vitamin and mineral supplements. The remaining 63 medications (26%) were either products used for pressure ulcer or other dermatological indications not included in the appropriateness list (17), or other medications (47) for which appropriateness had been considered neither by the Delphi panel nor by previous research [22, 23]. For statistical analyses, these 80 medications were grouped together and categorized as “other medications”.

Table 2 shows that, in this cohort, the proportion of residents exposed to the three categories of “generally”, “sometimes”, or “exceptionally” appropriate medications did not change much. There was a significant decrease in the total number of “sometimes” appropriate medications (from 194 pre to 167 post-intervention) and for “other” medications (from 31 to 21, not shown). There was, however, a significant reduction in the overall medication burden. The total number of regular medications decreased by 12.1%, from 372 at baseline to 327 at the end of follow-up (OR: 0.81; 95% CI: 0.70–0.92). The mean number of regular medications per participant decreased from 7.86 to 6.81 (*p* = 0.007)).

Mean levels of agitation did not change, six of the 44 participants showed severe agitation (score > 45) at baseline as compared to five residents with severe agitation among the 39 observed at follow-up. There was a slight yet statistically significant increase in the level of pain post-intervention (Table 2).

Odds ratios and corresponding 95% confidence intervals estimating the risk associated with having at least one prescription at the end the follow-up as compared to baseline were 0.82 (0.52–1.30), 0.83 (0.74–0.94), 1.16 (1.00–1.36) and 0.53 (0.42–0.67) for “exceptionally appropriate”, “sometimes-appropriate”, “generally appropriate”, and “other medications”, respectively (Table 3).

**Table 3:** Odds ratio for the risk of having a prescription pre versus post-intervention

Medication use among 44 participants	Odds ratio	95% CI
All medications	0.81	(0.71–0.92)
By appropriateness category^a^:		
“Generally appropriate” medication	1.16	(1.00–1.36)
“Sometimes appropriate” medication	0.83	(0.74–0.94)
“Exceptionally appropriate” medication	0.82	(0.52–1.30)
“Other” medication^b^	0.53	(0.42–0.67)

^a^According to the medication appropriateness list [23]

^b^“Other” medications comprised those for which the Delphi panel had not reached a consensus as well as the medications not considered in the appropriateness list [23]

Reduction in the use of antipsychotic agents however was minimal: detailed analyses showed that three antipsychotic agents were stopped, two were increased and for one the dosage was reduced. There were very few active prescriptions for cholinesterase inhibitors or memantine with only five prescriptions at baseline and four at the end of follow-up (Additional file 2).

The post-intervention KE session was attended by 22 health professionals, overall the intervention was positively evaluated (Table 4) In response to the open-ended questions, two thirds of the respondents mentioned inter-professional relations (e.g. team meetings, nurse involvement) as being very important, and one third indicated that information exchange had been clear and rigorous. Perceived barriers associated with medication review and adjustment included workload, difficulties in communication between shifts, staff turnover, and the fact that GPs and pharmacists are often off-site. Discontinuation of antipsychotic agents was identified as being difficult (Table 4).

**Table 4:** Evaluation of the intervention by health professionals attending the post-intervention KE session

#	Questions with multiple choice answers	Summary of responses
1	What do you think about the objective of optimizing medication for NH residents with severe dementia?	**All respondents** found that the study objective was a good idea.
2	Did you attend the first KE session?	**The majority of respondents** had participated in the first education session.
3	What about the relevance of the first KE session?	**All respondents** found the first education session either relevant or very relevant.
4	Had the first KE session influenced your attitude regarding the medication of residents with severe dementia?	The first education session influenced the attitude of **all but one respondent** who was already convinced of the merits of medication optimization.
5	Have you studied the provided MRG tool?	**Over half of the respondents** reported having read the provided MRG tool.
6	Has the MRG tool been useful in your practice?	**All respondents** found the provided MRG tool either useful or very useful.
7	How often did you use the MRG tool during the intervention period?	The degree of use of the MRG tool was **highly variable**, possibly depending on the respondents’ responsibilities and experience.
8	Did the study nurse interfere with your work?	**All respondents** agreed on the noninterference of the study nurse.
9	Was your workload increased by the intervention?	**More than half of respondents** did not notice an increase in their workload; only one mentioned a greatly increased workload.
10	Was the residents’ behavior changed by the intervention?	**More than half of respondents** did not observe changes in the residents’ behavior. One respondent mentioned positive changes for some patients but negative for others, another found it difficult to evaluate.
11	Was the quality of life of the residents changed by the intervention?	**None of the respondents** mentioned a deterioration of the quality of life of residents.
12	Do you feel that NH staff should be sensitized to the complexity of medication for the residents with severe dementia?	**There was unanimity** on the need to educate NH staff regarding this issue.
13	Do you feel that NH staff should receive more information regarding the medication of residents with severe dementia?	**The majority of respondents** agreed, only two respondents were not sure.

## Discussion

Medication revision where consideration is given to reducing less appropriate medications for NH residents suffering from severe dementia, which underlies the present research, stems from the desire to provide comfort care in the presence of severe dementia [3, 21, 22, 32–34]. This approach is not based on an estimate of remaining life expectancy, but on the consideration that residents with severe dementia benefit more from increased comfort than from increased life expectancy [22]. Medication appropriateness categories, as presented in the medication review guidance tool, should and do not replace clinical judgement by the resident’s physician and care team, who would take all clinical, psychological and social characteristics of the resident into account. This idea is reflected by the terms used: the Delphi panel members expressed the necessity to always make individualized therapeutic decisions and were unable to retain a “never” nor an “always” category [23]. The idea of considering certain medications, beyond criteria on potentially inappropriate medications for seniors such as those by Beers [19], as only “sometimes” or “exceptionally” appropriate for seniors suffering from severe dementia, is based on the conviction that these seniors should benefit from a comfort care approach [22, 32]. The clinical presentation of severe dementia, as indicated by the Reisberg scale [35], should trigger a change towards this care approach. When the risk to benefit ratio is in doubt (e.g. maintaining warfarin in the presence of atrial fibrillation for secondary stroke prevention with the need for frequent monitoring of blood level posing a heavy burden), it is the physician’s clinical judgement determining whether to continue or deprescribe. These decisions are difficult and must include the family, if available, the care team, and all information regarding the senior’s well-being.

The aim of this study was to establish the feasibility and acceptability of an interdisciplinary knowledge-exchange intervention to reduce medication load for NH senior residents with severe dementia. Our results indicate that the intervention was feasible and well accepted by health care professionals. The overall reduction in the number of medications per resident (12%) is encouraging as it may translate to less discomfort related to medication taking and monitoring and to time savings for care staff.

As shown in Additional file 2, these reductions concerned most significantly the category of “sometimes” appropriate medications, notably antidiabetic (from 12 to 7 prescriptions, 42%), antihypertensive (from 28 to 21, 25%), antidepressant (from 19 to 16, 16%) and laxative medications (from 46 to 42, 9%). Multivitamins, a medication class for which no Delphi consensus was achieved, were reduced from 21 to 7 prescriptions (67%). As for psychotropic drugs, a very small decrease in the number of regular prescriptions for antidepressants and antipsychotics was observed. There was no modification in the number of regular prescriptions for anxiolytic agents, which were considered “generally” appropriate [22, 23]. Thus, the OptimaMed intervention did not have a meaningful impact on the use of psychotropic drugs, but the study took place in settings already sensitive to the considerable risk of serious adverse effects associated with these medications [36–39]. It is interesting to note that the baseline average of 8 regular prescriptions per participating resident was lower than the 2012 Canadian NH average of 10 or more different medications in this population [11]. Despite low baseline medication use, a further reduction in medication load and in the use of only “sometimes appropriate” medications was observed.

Some NH antipsychotic deprescribing studies have generated encouraging results [40, 41] although one study did report an increase in neuropsychiatric symptoms in the intervention group [42]. Further interventions emphasize deprescribing of antipsychotics for neuropsychiatric symptoms of dementia. In Canada, this is facilitated by the recently published guidelines for the deprescribing of antipsychotics in dementia [43]. Given that at the time of this study, in 2014, these or other guidelines specific to the Canadian context had not yet been published, we were unable to include them in our pilot intervention. Study physicians had to follow prior available clinical guidance to progressively deprescribe certain medications, such as antipsychotics, for which a slight reduction was observed.

Previous deprescribing interventions have concluded that it is relatively safe to deprescribe antihypertensives (including diuretics), statins, and benzodiazepines in seniors [16, 44, 45]. In the present study, we observed no changes in the level of agitation, but a statistically significant increase in the measured level of pain. This increase of 1.6 point (the PACSLAC-F scale ranging from 0 to 60 points), over 4 months is not clinically worrisome, however, particularly in the context of the evolution of severe dementia.

Our intervention built upon the promising work by Garfinkel and colleagues, who incorporated evidence for medication indication, effectiveness, and adverse effects as well as patient circumstances and continuation preferences in an algorithm to improve drug therapy in frail seniors [46, 47]. In a Dutch cluster randomized trial to discontinue inappropriate medications, physicians in collaboration with pharmacists performed one multidisciplinary, multistep medication review for NH residents [48]. After a mean follow-up of 144 days, at least one inappropriate medication (according to the STOPP criteria [49]) was discontinued for 39% of participants, as compared to 29.5% in the control group, for an adjusted relative risk of 1.23 (95% CI 1.02–1.75), while there was no deterioration of clinical outcomes. In a randomised controlled trial on deprescribing, the intervention group had a mean reduction of 1.9 (SD 4.1) medications compared to an increase of 0.1 (SD 3.5) in the control group, for an estimated difference of 2.0 (95% CI 0.08–3.8) without significant differences for other outcomes [50]. However, none of these studies specifically addressed the particularly vulnerable subgroup of NH residents with severe dementia who may necessitate more specific criteria of medication appropriateness [22].

To our knowledge, our study is the first intervention based on a medication review guidance tool developped in Canada for NH seniors with advanced dementia. There are several limitations, however, that apply to the results of this study which must be interpreted cautiously. First, using a quasi-experimental design. it was not possible to determine whether the observed increase in the mean level of pain was a consequence of the intervention or of the evolution of the disease. Second, the sample size was too small to allow for adjustments for potential confounders. Third, the short follow-up duration did not permit to evaluate how sustainable the observed effects would be in the long run. On the other hand, the life expectancy of NH senior residents with severe dementia is limited, indeed 11% of our participants died during the 104-day period. Fourth, due to the NH GPs and pharmacists limited availability, the interval between the medication review and the measurement of outcomes varied between participants. Fifth, we were able to evaluate the levels of pain and of agitation at baseline and at the end of follow-up only. The same well-trained nurse performed pre and post measurements with the help of validated tools, but those were snapshots of behaviours likely to fluctuate from day to day. Finally, with the aim of testing the feasibility of implementing the OptimaMed intervention in Quebec NHs, we did not document the clinical decision-making process itself nor the specific reasons why medications were continued or deprescribed.

Several barriers and facilitators, identified by the NH care teams, may be addressed to improve the intervention. Adding specific information regarding the challenges of neuropsychiatric symptoms to KE sessions may prove useful. In addition to providing families with medication use information, it was also suggested that it would be important to consider further involvement of families in discussions regarding medication use. Research on challenges regarding KE with families and the ethical aspects of adjusting medication is ongoing, [51–53] and may help improve shared decision making in NHs. The quality of information exchange within the care team was identified as being a critical barrier, but workload, staff turnover and the limited availability of GPs and pharmacists were also mentioned as organisational/structural barriers. Improvements in those matters would, however, require changes in governmental policies.

## Conclusions

This quasi-experimental study tested the feasibility of an interdisciplinary intervention comprising KE and a tool to facilitate medication review for NH senior residents with severe dementia. Its results are encouraging with regard to reduction of overall medication burden, feasibility and NH staff interaction. The OptimaMed intervention may have the potential to improve medication use among this particularly vulnerable population of seniors. Ongoing regulatory changes regarding the roles and autonomy of pharmacists and nurses in North America, including the province of Quebec, may further this intervention. The conduct of clinical examinations by nurses and the adjustment of medication dosage by pharmacists may palliate the limited availability of GPs in NHs, and provide for a more harmonious work flow between all health care professionals.

## Additional files

Additional file 1: Medication appropriateness list. (DOCX 37 kb)

Additional file 2: Number of all regular medications, according to the medication appropriateness list [1] and the WHO-ATC classes. (DOCX 30 kb)

## Abbreviations

AHFS: American Hospital Formulary Service
CIUSSSCN: *Centre intégré universitaire de santé et de services sociaux de la Capitale-Nationale*
CI: Confidence Intervals
CMAI: Cohen-Mansfield Agitation Inventory
FAST: Functional Assessment Staging Test
GLMM: Generalized Linear Mixed Models
GP: General Practitioner
HSSB: Health and Social Services Board
KE: Knowledge Exchange
MRG: Medication Review Guidance
NH: Nursing Home
OR: Odds Ratio
*p*: Probability
PACSLAC-F: Pain Assessment Checklist for Seniors with Limited Ability to Communicate-French
SD: Standard Deviation
SMAF: *Système de Mesure de l’Autonomie Fonctionnelle*
STOPP/START: Screening Tool Of Older People’s Prescription/Screening Tool To Alert To Right Treatment
