# Supplementary material for: The OptimaMed intervention to reduce inappropriate medications in nursing home residents with severe dementia: results from a quasi-experimental feasibility pilot study
Source: BMC Geriatr. 2018 Sep 4;18:204. doi: 10.1186/s12877-018-0895-z (PMC6123948; doi:10.1186/s12877-018-0895-z)
Supplement: Supplementary file 1 — Medication appropriateness list. (DOCX 37 kb) [file 12877_2018_895_MOESM1_ESM.docx]

**Additional file 1** Medication appropriateness list

|  | | ATC code | | | Medication class | |
| --- | --- | --- | --- | --- | --- | --- |
| **GENERALLY** | | D01A | | | Antifungals for topical use | |
|  |  | N02A | | | Opioid analgesics | |
|  |  | N02BE01 | | | Other analgesics and antipyretics: Acetaminophen | |
|  |  | N03A | | | Antiepileptics | |
|  |  | N05B | | | Anxiolytics | |
|  |  | R03AC | | | Adrenergics, inhalants | |
|  |  | S01XA20 | | | Artificial tears | |
|  | ATC code | | | Medication class | |  |
| **EXCEPTIONALLY APPROPRIATE MEDICATIONS** | B01A | | | | Antithrombotic agents, excluding acetylsalicylic acid | |
|  | C01AA05 | | | | Cardiac therapy: Digoxin | |
|  | C01B | | | | Antiarrhythmic agents, class I and III | |
|  | C02AC01 | | | | Antihypertensives: Clonidine | |
|  | C02D | | | | Antihypertensives acting on arteriolar smooth muscles | |
|  | C10A | | | | Lipid modifying agents | |
|  | G03 | | | | [Sex hormones and modulators of the genital system](http://www.whocc.no/atc_ddd_index/?code=G03) | |
|  | G04BD | | | | Drugs for urinary frequency and incontinence | |
|  | L01 | | | | Antineoplastic agents | |
|  | L02B | | | | Hormone antagonists and related agents | |
|  | L04A | | | | Immunosuppressants | |
|  | M01A | | | | Anti-inflammatory and antirheumatic products, non-steroids | |
|  | M04AC01 | | | | Antigout preparations: colchicine | |
|  | M05B | | | | Drugs affecting bone structure and mineralization | |
|  | N05BB01 | | | | Anxiolytics: hydroxyzine | |
|  | N05CM05 | | | | Hypnotics and sedatives: Scopolamine | |
|  | N06AA | | | | Antidepressants, non-selective monoamine reuptake inhibitors | |
|  | | | | | | |
|  | | |  | | | |
| ATC code | | | Medication class | | | |
| **SOMETIMES APPROPRIATE MEDICATIONS** | R03BB | | | | Anticholinergics for obstructive airway diseases | |
|  | R03DC | | | | Leukotriene receptor antagonists | |
|  | R06A | | | | Antihistamines for systemic use, first generation | |
|  | A02BC | | | | Proton-pump inhibitors | |
|  | A06A | | | | Drugs for constipation | |
|  | A07D | | | | Antipropulsivants | |
|  | A10 | | | | Drugs used in diabetes | |
|  | B01AC06 | | | | Platelet aggregation inhibitors: Acetylsalicylic acid | |
|  | C01DA | | | | Vasodilators used in cardiac disease | |
|  | C03 | | | | Diuretics | |
|  | C07A | | | | Beta blocking agents | |
|  | C08 | | | | Calcium channel blockers | |
|  | C09 | | | | Agents acting on the renin-angiotensin system | |
|  | G04CA | | | | Antagonist of α-adrenoreceptors in the prostate and bladder | |
|  | H02 | | | | Corticosteroids for systemic use | |
|  | H03 | | | | Thyroid therapy | |
|  | J01 | | | | Antibacterials | |
|  | J05A | | | | Antivirals | |
|  | M04A | | | | Antigout preparations | |
|  | N05A | | | | Antipsychotics | |
|  | N06A | | | | Antidepressants, excluding tricyclic antidepressants | |
|  | P03A | | | | Ectoparsiticides | |
|  | R01AD | | | | Corticosteroids in nasal preparations | |
|  | [R06A](http://en.wikipedia.org/w/index.php?title=ATC_code_R06AA52&action=edit&redlink=1) | | | | Antihistamines (2nd generation), also as antiemetics | |
|  | S01B | | | | Ophthalmological anti-inflammatory agents | |
|  | S01E | | | | Antiglaucoma preparations | |

1. Kröger E, Wilchesky M, Marcotte M, et al. Medication Use Among Nursing Home Residents With Severe Dementia: Identifying Categories of Appropriateness and Elements of a Successful Intervention. J Am Med Dir Assoc. 2015;16(7):629.e1-17.
