# Supplementary material for: The OptimaMed intervention to reduce inappropriate medications in nursing home residents with severe dementia: results from a quasi-experimental feasibility pilot study
Source: BMC Geriatr. 2018 Sep 4;18:204. doi: 10.1186/s12877-018-0895-z (PMC6123948; doi:10.1186/s12877-018-0895-z)
Supplement: Supplementary file 2 — Number of all regular medications, according to the medication appropriateness list [1] and the WHO-ATC classes. (DOCX 30 kb) [file 12877_2018_895_MOESM2_ESM.docx]

**Additional file 2** Number of all regular medications, according to the medication appropriateness list[[1](#_ENREF_1)] and the WHO-ATC classes

| **ATC code** | Medication class | **Number of regular prescriptions** | |
| --- | --- | --- | --- |
|  |  | **pre intervention** | **post intervention** |
| **generally appropriate medications** | | | |
| N02A | Opioid analgesics | 19 | 32 |
| N02BE01 | Other analgesics and antipyretics: Acetaminophen | 20 | 19 |
| N03A | Anti-epileptics | 4 | 3 |
| N05B | Anxiolytics (except for hydroxyzine) | 35 | 36 |
| R03AC | Medications for obstructive airway diseases (adrenergic inhalers) | 2 | 1 |
| S01XA20 | Artificial tears | 7 | 7 |
| **sometimes appropriate medications** | | | |
| R03B | Medications for obstructive airway diseases (non adrenergic inhalers) | 2 | 1 |
| A02BC | Proton pump inhibitors | 15 | 13 |
| A06A | Laxatives | 46 | 42 |
| A10 | Drugs used in diabetes | 12 | 7 |
| B01AC06 | Platelet aggregation inhibitor: acetylsalicylic acid (ASA) | 12 | 10 |
| C01 | Vasodilators | 3 | 2 |
| C03 | Diuretics | 4 | 3 |
| C05AA01 | Hydrocortisone | 0 | 1 |
| C07A | Beta blocking agents | 9 | 7 |
| C08 | Calcium channel blockers | 7 | 5 |
| C09 | Agents acting on the renin-angiotensin system | 5 | 4 |
| G04C | Drugs used in benign prostatic hypertrophy | 7 | 7 |
| H03AA01 | Thyroid hormones | 15 | 15 |
| J01 | Antibacterials | 2 | 1 |
| N05A | Antipsychotics | 27 | 26 |
| N06A | Antidepressants^*^ | 19 | 16 |
| S01 | Ophthalmological preparations (antibacterials and anti-glaucoma) | 11 | 10 |
| **exceptionally appropriate medications** | | | |
| B01A | Antithrombotic agents, excluding ASA | 2 | 3 |
| C10A | Lipid modifying agents | 2 | 1 |
| L01 | Antineoplastic agent | 1 | 1 |
| M05B | Drugs affecting bone structure and mineralization | 2 | 1 |

| **ATC code** | Medication class | **Number of regular prescriptions** | |
| --- | --- | --- | --- |
|  |  | **pre intervention** | **post intervention** |
| **medications for which no Delphi consensus was achieved** | | | |
| A11 | Multivitamins | 21 | 7 |
| A12AA | Calcium | 7 | 5 |
| B03B | Vitamin B_12_ and folic acid | 3 | 1 |
| D06A | Topical antibiotic | 1 | 0 |
| N06D | Cholinesterase inhibitors and memantine | 5 | 4 |
| **other medications** | | | |
| A02AF | Antacids with antiflatulents | 1 | 1 |
| A03FA03 | Propulsives | 2 | 2 |
| D0 | Dermatologicals, except topical antibiotics | 30 | 26 |
| M02 | Topical products for joint and muscular pain | 5 | 3 |
| M03 | Muscle relaxants | 1 | 0 |
| N04B | Dopaminergic agents for Parkinson disease | 3 | 1 |
| N05CD07 | Hypnotics/sedatives: temazepam | 1 | 1 |
| R01AX1 | Other nasal preparations | 0 | 1 |
| R05D | Cough and cold preparation: codeine | 1 | 0 |
| S01 | Other ophthalmological preparations (except for antibacterials and anti-glaucoma listed above) | 1 | 1 |
| **Total number** | | **372** | **327** |

^*^ This group comprises no tricyclic antidepressants nor non-selective monoamine reuptake inhibitors (N06AA).

**Reference**

1. Kröger E, Wilchesky M, Marcotte M et al. Medication Use Among Nursing Home Residents With Severe Dementia: Identifying Categories of Appropriateness and Elements of a Successful Intervention. J Am Med Dir Assoc. 2015;16(7):629.e1-17.
